# Quantifying Fish Assemblages in Large, Offshore Marine Protected Areas: An Australian Case Study

**DOI:** 10.1371/journal.pone.0110831

**Published:** 2014-10-31

**Authors:** Nicole A. Hill, Neville Barrett, Emma Lawrence, Justin Hulls, Jeffrey M. Dambacher, Scott Nichol, Alan Williams, Keith R. Hayes

**Affiliations:** 1 Institute for Marine and Antarctic Studies, University of Tasmania, Hobart, Tasmania, Australia; 2 Digital Productivity Flagship, Commonwealth Scientific and industrial Research Organisation (CSIRO), Brisbane, QLD, Australia; 3 Digital Productivity Flagship, Commonwealth Scientific and industrial Research Organisation (CSIRO), Hobart, Tasmania, Australia; 4 Geoscience Australia, Canberra, ACT, Australia; 5 Oceans and Atmosphere Flagship, Commonwealth Scientific and industrial Research Organisation (CSIRO), Hobart, Tasmania, Australia; University of Glasgow, United Kingdom

## Abstract

As the number of marine protected areas (MPAs) increases globally, so does the need to assess if MPAs are meeting their management goals. Integral to this assessment is usually a long-term biological monitoring program, which can be difficult to develop for large and remote areas that have little available fine-scale habitat and biological data. This is the situation for many MPAs within the newly declared Australian Commonwealth Marine Reserve (CMR) network which covers approximately 3.1 million km^2^ of continental shelf, slope, and abyssal habitat, much of which is remote and difficult to access. A detailed inventory of the species, types of assemblages present and their spatial distribution within individual MPAs is required prior to developing monitoring programs to measure the impact of management strategies. Here we use a spatially-balanced survey design and non-extractive baited video observations to quantitatively document the fish assemblages within the continental shelf area (a multiple use zone, IUCN VI) of the Flinders Marine Reserve, within the Southeast marine region. We identified distinct demersal fish assemblages, quantified assemblage relationships with environmental gradients (primarily depth and habitat type), and described their spatial distribution across a variety of reef and sediment habitats. Baited videos recorded a range of species from multiple trophic levels, including species of commercial and recreational interest. The majority of species, whilst found commonly along the southern or south-eastern coasts of Australia, are endemic to Australia, highlighting the global significance of this region. Species richness was greater on habitats containing some reef and declined with increasing depth. The trophic breath of species in assemblages was also greater in shallow waters. We discuss the utility of our approach for establishing inventories when little prior knowledge is available and how such an approach may inform future monitoring efforts within the CMR network.

## Introduction

Marine ecosystems face pressures from a range of sources, including pollution, fishing, habitat destruction, invasive species and a changing climate [1]. These pressures threaten the essential processes and resources that marine ecosystems provide, such as climate regulation, nutrient cycling and the provision of food, as well as threatening their intrinsic natural and cultural value [2]. As a consequence, management strategies have been adopted to safeguard marine ecosystems and their associated biodiversity. These strategies include the establishment of Marine Protected Areas (MPAs) which offer varying levels of protection according to their zoning or IUCN designation [3]. Countries such as the United States, Australia, South Africa, New Zealand, Kenya and the Philippines have a long history of establishing MPAs [4], [5]. However, the absence of a truly global system of MPAs prompted the Convention of Biological Diversity [6] and the Jakarta Mandate [7] to provide a framework and renewed international impetus for protecting marine biodiversity. This framework has resulted in a significant increase in recent years in the declaration of MPAs worldwide, with many forming part of large networks or systems of MPAs [8].

The increasing global commitment to the sustainability of healthy marine ecosystems is encouraging, but the declaration alone of MPAs does not ensure the conservation of biodiversity or ecosystems [4]. Management authorities must: clearly articulate the objectives of individual MPAs and MPA networks, both of which may contain multiple IUCN zones; formulate and enforce management plans [9] and periodically revisit and evaluate their management plans, which in turn requires that they establish a long-term biological monitoring program [4]. Developing monitoring programs for individual MPAs within networks of MPAs is often a difficult task. There is generally a mismatch between the spatial scales of information used to design MPA networks and those required to manage individual MPAs. Inventories of the types and distribution of habitats, communities and species within individual MPAs are often required before monitoring programs can be designed. In addition, for offshore or difficult to access MPAs, there are significant logistical and statistical challenges to working in remote environments.

Australia, a signatory to the CBD, provides a good example of the challenges involved in ultimately developing long-term monitoring programs in large MPA networks. In 2012, the Australian government announced an expansion of its MPA network to include 33 new MPAs. This makes a total of 60 MPAs covering approximately 3.1 million square kilometres, divided between six planning bioregions [10]. Although called the “Commonwealth Marine Reserve” or “CMR” network, areas with IUCN zones ranging from 1a (Sanctuary Zone) to VI (General Use Zone) are distributed throughout the network. From this point forward we adopt the above terminology and refer to MPAs within this network as reserves regardless of their IUCN zoning. All of the reserves in the CMR are however, located in offshore waters, most are large and encompass a broad depth range, and many are remote.

The Australian CMR network aims to meet the principles of being Comprehensive, Adequate and Representative (the CAR principles) [11] and utilised the national Integrated Marine and Coastal Regionalisation of Australia (IMCRA; [12]). The IMCRA bio-regionalisation is based on breaks in the distribution of demersal fish communities (together with some physical datasets) and is the best available continental-scale regionalisation of Australian marine fauna. Hence it was the most appropriate mechanism for delineating management regions and informing the placement of reserves. When designing biological monitoring programs for individual reserves however, much finer scale information is needed on the spatial distribution of important habitats (such as reefs) and the biological communities they contain. For many reserves in the new network this level of information is simply unavailable. It is therefore necessary to provide a robust inventory of the biological communities and key species within the reserves, delineate their spatial distribution and quantify the relationship between these distributions and key environmental drivers to enable the development of monitoring programs that will ultimately evaluate the effectiveness of each reserve.

Inventory, and ultimately monitoring, of large, deep reserves is challenging and relatively expensive. It requires vessels large enough to conduct surveys safely in offshore waters, together with a suite of non-extractive survey methods that can be deployed at depth, and sampling designs that are efficient and flexible enough to accommodate multiple (and potentially changing) objectives. A suite of non-extractive survey methods are currently available including multibeam sonar (MBS) for characterising the seafloor and associated habitat types [13], and image-based methods, such as towed camera systems, cameras attached to autonomous or remotely operated vehicles and baited cameras, for observing benthic fauna. Here we trialled MBS and Baited Remote Underwater Video (BRUVs), deployed within a spatially balanced design, for inventorying demersal fish in the multiple use zone (IUCN VI) of one reserve in the Australian network, the Flinders CMR. We use BRUVs because of their demonstrated ability to survey fish in deep waters [14] and because they have been extensively used in Australian inshore environments for monitoring and other ecological applications [15]–[17]. We trialled a spatially-balanced (i.e. evenly spread out in space) design known as the Generalised Random- Tessellation Stratified (GRTS) design [18] because it is flexible and provides a good way to obtain a representative sample that respects the spatial distribution of habitats and communities in the target population [19].

In this study we: identify patterns in demersal fish assemblages across the continental shelf (a multiple-use zone, IUCN VI) of the Flinders CMR; quantify these patterns in relation to environmental gradients; examine the spatial distribution of assemblages across the reserve; and describe how our approach and the information gained may be useful to the development of a long-term biological monitoring program. We focus on fish communities because: broad-scale distributional records of demersal fish were instrumental in the marine bioregionalisation that underpins the design of the reserve network [20]; many species are of recreational and commercial value and are therefore likely to respond to current and future management actions within the reserve; and demersal fish are key components of benthic ecosystems.

## Methods

### Survey Area

**Figure 1:**
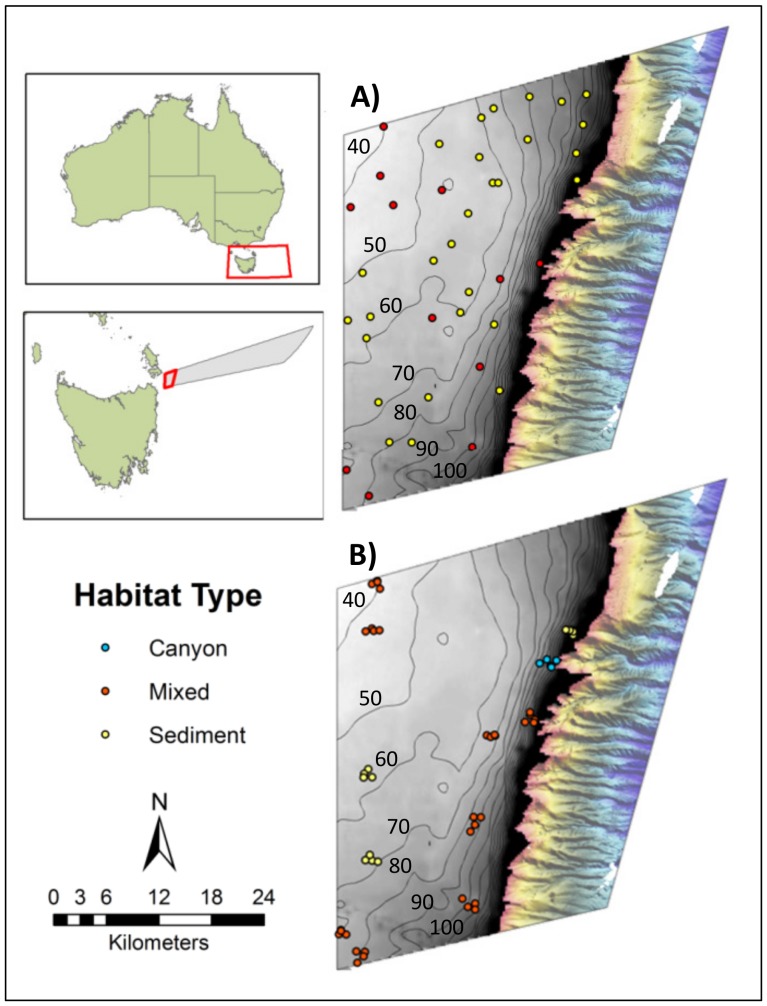
Location of the Flinders Commonwealth Marine Reserve (CMR) in Tasmania, Australia. Panels A and B show the CMR's Multiple Use Zone (IUCN VI) where survey work was conducted. The grey zone is the continental shelf (less than 200 m depth) where there was little pre-existing mapping data. Coarse bathymetry data (gridded at 250 m horizontal resolution) sourced from Geoscience Australia is shown with 10 m contour intervals overlain. The coloured area to the right shows the relatively steep and highly incised upper continental slope that had been mapped previously with multibeam sonar extending from 200 to 1500 m. A) Location of the 40 sites surveyed for habitat type in phase one of the sampling program. B) Location of the clustered sites where Baited Remote Underwater Videos (BRUVs) were deployed in phase two. Sites are coloured according to the broad habitat type: sediment (yellow); mixed, low-profile reef and sediments (red); canyon head (blue) recorded during phase one of sampling.

The Flinders Commonwealth Marine Reserve (CMR) was established in 2007 and lies about 25 km offshore of northern Tasmania (Fig. 1). The reserve is 26,975 km^2^ in size and extends as a west-east corridor from 35 m to > 3,000 m water depths. It consists of two zones: a multiple use zone (IUCN Category VI) that covers the majority of the continental shelf and slope; and a marine national park zone (IUCN Category II) that extends from the continental slope to the edge of the reserve at the 200 nautical mile limit of Australia's Exclusive Economic Zone (Fig. 1 inset). Our study area is the continental shelf, part of the multiple use zone (IUCN Category VI), where activities that impact on benthic habitats are prohibited, including demersal trawling, Danish seining and scallop dredging, or subject to permit requirements, for example mining activities [21]. Benthic habitats on the Flinders CMR shelf consist of sediment plains with patches of low profile and sand-inundated reefs, and steep rocky outcrops where canyon heads incise the shelf break (Lawrence, unpublished data). Within this environment, reefs and rocky outcrops have been identified as features likely to contain enhanced benthic diversity [21].

The Flinders CMR shelf straddles two provincial biogeographical regions: the Southeast Shelf Transition in the north and the Tasmanian Shelf Province in the South [12]. This region is characterised by variable but generally high exposure and strong tidal currents, especially in shallow areas between Flinders Island and the Tasmanian mainland [22], [23]. The region is also influenced by southwards incursions of the East Australian Current (EAC) which brings warmer waters on to the shelf in summer [24]. The flora of the region is moderately rich and contains species common in cold temperate waters as well as low abundances of species common to warmer temperate waters [20].

### Sampling design and methods

In contrast to the Flinders CMR continental slope, which has been comprehensively mapped using multibeam sonar (from which seafloor habitats have been inferred), very little spatially explicit habitat or biological information is available for the shelf. This represents a challenge for designing targeted sampling programs. Interpolated bathymetry for the Flinders shelf exists, but is at a gridded resolution of 250 m [25] (Fig. 1) which does not allow the identification of fine scale features of interest on the shelf, such as reefs. As a consequence of our limited *a priori* knowledge of the area, the inventory and description of fish assemblages presented here forms one component of a multi-objective and multi-phase study within the Flinders CMR. Additional objectives of the broader study include examining the distribution and estimating the area of the different habitat types within the reserve, as well as quantifying benthic invertebrate communities using imagery captured by towed video and AUV, and these results will be reported elsewhere. To accommodate the objectives of the different components of the study, we used the probabilistic design, Generalised Random –Tessellation Stratified sampling technique (GRTS; [18]). GRTS ensures sampling sites are well spread out across the survey area (spatially-balanced), a desirable property for spatial sampling that enhances estimation efficiency and representativeness [18]. Other common sampling designs include simple or stratified random sampling or systematic sampling. However, simple random sampling often clumps sites [19] and stratified random sampling (based on habitat in our case) was not possible without more detailed knowledge of the study area. Systematic sampling prevents clumping but an accurate design-based variance estimator does not exist; a factor that was important for other aspects of the broader study [18], [26]. provide a good discussion of the relative merits of various spatial sampling strategies. Another property of GRTS is that it produces an ordered list of sample sites and any set of consecutive sampling sites also maintain spatial balance, offering flexibility to adaptively change sample sizes in the field, if for example, sampling takes less time than expected. To date GRTS has primarily been used in natural resource assessment and monitoring in the United States e.g. [27].

Because of our lack of knowledge on the distribution of habitat types, we implemented a two-phase sampling program. In phase one, the distribution of habitat types across the reserve was quantified by characterising the first forty, 200 m square, sites from a master list of GRTS sites (essentially an ordered list of all possible GRTS sites in the study area. See [28] for a detailed discussion on the use of master lists) as either ‘soft’ sediments or ‘mixed’, low profile patchy reef using MBS and footage obtained from a drop camera (Fig. 1; Lawrence, unpublished data). In phase two of the sampling program, the subject of this paper, we sampled the fish assemblages using BRUVs. Ideally we would have revisited all forty of the phase one GRTS sites. The large size of the study area and the soak time required for each BRUV deployment however, meant that it was not possible to do so in the ship time available. Instead, we sampled clusters of sites surrounding a subset of the phase one GRTS- sites. In phase two, we sampled mixed reef habitats more intensively than sediment habitats because shelf reef systems within the reserve are recognised as an important biodiversity feature and a *priori* we expected that reefs would harbour a greater diversity of assemblage types than sediments. We utilised the fact that consecutive GRTS sites maintain spatial balance and selected (from the ordered list of phase one sites) the first eight mixed habitat sites and the first three sediment sites of the forty phase one sites as the basis of the phase two clusters (Fig. S1. shows the site numbers of the forty phase ones sites and lists those used as the basis of phase two clusters.) This amounts to selecting phase one sites for the BRUV sampling that are spatially balanced *within* each of the phase one habitat types, mixed reef and sediments, and presumably therefore also spread across the environmental gradients in the region. In addition, a site was added near the head of a canyon, to ensure representation of another identified feature of the reserve which occupies a relatively small proportion of space and is therefore less likely to be sampled in a probabilistic sampling design (Fig. 1B). Clusters of sites within 1 km of the subset of phase one sites were selected again using the GRTS master list, resulting in five sites per cluster (Fig. 1B).

At each site, one BRUV was deployed using systems and deployment conditions widely used in Australia [29]. Each stereo-BRUV consisted of a frame that houses a stereo camera pair, a bait bag attached to an arm within field of view of the cameras, a diode for synchronising imagery between camera pairs. In water deeper than approximately 70 m, a light with a blue filter was also attached [15]. Stereo- BRUVs were baited with 1 kg of crushed pilchards and deployed for 60 min (soak time). Appropriate ethics (University of Tasmania Animal Ethics Permit: A12514) and fieldwork (Australian Government Director of National Parks Approval of Research Activities in the Southeast Commonwealth Marine Reserve Network. Ref No 07/10622) approvals were obtained for this work.

### Analysis

Imagery collected using stereo-BRUVs was scored using standard metrics including scoring the maximum number of fish occurring in any one frame for each species (MaxN) [15], [30]. Scoring was completed with Event Measure software [31].

To determine the types of fish assemblages present at sites sampled on the Flinders CMR shelf, fuzzy clustering was performed on multivariate fish composition data using the ‘cluster’ package [32] in R [33]. Multivariate data were square root transformed to reduce the influence of the most abundant species and a Bray Curtis dissimilarity matrix generated. The number of fuzzy clusters was set to six, following preliminary analysis using several different clustering methods, and the membership exponent (which controls the ‘fuzziness’ of clusters) set to 1.3 after examining silhouette profiles of group membership.

A constrained ordination (Canonical Analysis of Principle Co-ordinates; CAP) [34] was used to test if the assemblage groups determined by fuzzy clustering were significantly different from each other (using a permutational test). In addition, jack-knife sampling was used to examine the overall and individual classification accuracy of the six groups. To enable the ecological interpretation of assemblages, the trophic level and habitat preference of species was defined. Species were assigned one of seven habitat preference categories: 1) pelagic; 2) wide-ranging demersal; 3) sediment associated; 4) reef and sediment associated; 5) reef and seagrass associated; 6) reef associated; and 7) demersal generalists (found in more than two habitats) based on species attributes described in [35], [36]. The trophic level of species was extracted from FishBase [37]. Differences in the trophic level of species comprising the six groups was assessed with a one factor Analysis of Variance (ANOVA) after transforming data to satisfy the assumptions of ANOVA. To understand which species were the key contributors to each of the six assemblage groups, a SIMPER analysis was performed. SIMPER decomposes the Bray-Curtis dissimilarity matrix between all pairs within a group into the percentage contributions from each species [34].

Correlations between fish assemblages and their environment were examined against three easily quantifiable environmental variables that, from the literature, may influence benthic assemblages in our system; latitude, depth and the substratum type. At each cluster of five sites, only the central site had been surveyed with MBS and drop camera in phase one of the sampling program. Therefore, substratum type was derived from the BRUV field of view and characterised as either: ‘sediment’ if no hard substratum or no hard substratum-associated organisms (i.e. sponges etc.) were present; ‘mixed’ if some hard substratum was visible or if some hard substratum-associated organisms were present; and ‘reef’ if the majority of the field of view contained hard substratum or hard substratum-associated organisms. Latitude was taken from the recorded location of BRUV drops and depth was derived using the 250 m resolution bathymetry grid available from Geoscience Australia [25]. Relationships between assemblage groups and environmental variables were inferred by examining correlations with CAP axes and overlaying vectors of the environmental variables on CAP plots. Ordination and SIMPER analysis were performed in PRIMER + with PERMANOVA [34].

The influence of environmental variables in determining patterns in species richness (the number of species present) was examined using Generalised Linear Mixed Models (GLMMs). Observations were modelled using a Poisson distribution with a log link function and sampling cluster was included as a normally distributed random factor. Mixed models were fit in R [33] with penalised quasi-likelihood, using the glmmPQL function in the package MASS [38].

## Results

### Distribution of habitats across the CMR

The results of phase one sampling indicated that the majority (70%) of the 40 sites on the shelf were sedimentary habitats, and the remainder ‘mixed’ reef and sediment habitat. Mixed reef habitat occurred in the north-west, south-west, and near the edge of the shelf of the study area (Fig. 1A; Lawrence, unpublished data).

### Fish assemblages present in the CMR

Sixty BRUV deployments were completed in phase two, of which 51 yielded video footage of sufficient quality for scoring fish assemblage composition. Overall, 45 species were observed, with a total of 1,837 individuals recorded (at Max N) (Table 1). Of these species, 66% are endemic to Australia. Leatherjackets (Monacanthidae) were the most numerically abundant group, and comprised mostly *Thamnaconus degeni* (Degan's leatherjacket) and *Meuschenia scaber* (velvet leatherjacket) with, respectively, a total of 471 and 97 individuals recorded and MaxN of 89 and 11. *Nemadactylus macropterus* (jackass morwong), and *Caesioperca Lepidoptera and C. razor* (butterfly and barber perch) were the next most abundant species (379 and 140 individuals recorded and MaxN of 89 and 60). Other abundant species included *Latris lineata* (striped trumpeter), *Parequula melbournensis* (silverbelly), *Pseudolabrus rubicundus* (rosy wrasse), *Helicolenus percoides* (reef ocean perch), and *Cephaloscyllium laticeps* (draughtboard shark). The five most prevalent species were *N. macropertus* (59% of sites), *C. laticeps* (57% of sites), *M. scaber* (53% of sites), *Neosebastes scorpaenoides* (common gurnard perch; 41% of sites) and *T. degeni* (41% of sites).

**Table 1:** List of species recorded in Baited Remote Underwater Video deployments across the continental shelf in the Flinders Commonwealth Reserve.

			Across CMR			Mean (SE) MaxN by group					
Family	Species name	Common name	Total MaxN	Max MaxN in any drop	Prevalence (% drops)	Group 1	Group 2	Group 3	Group 4	Group 5	Group 6
*Blenniidae, Gobiidae, Tripterygiidae*	*Blenniidae, Gobiidae, Tripterygiidae*	Blennies, Gobies, Triplefins	7	3	10	0.38 (0.18)	0.4 (0.31)	0	0	0	0
*Callorhinchidae*	*Callorhinchus milii*	Elephant shark	1	1	2	0	0.1 (0.1)	0	0	0	0
	*Nemadactylus douglasii*	Grey morwong	7	2	10	0	0.2 (0.13)	0.33 (0.24)	0.33 (0.33)	0	0
	*Nemadactylus macropterus*	Jackass morwong	379	43	59	0	10.7 (2.04)	23.22 (4.78)	0	5.25 (1.23)	0
*Cyttidae*	*Cyttus australis*	Silver dory	6	1	12	0	0.1 (0.1)	0	0	0.42 (0.15)	0
*Diodontidae*	*Diodon nicthemerus*	Globe fish	1	1	2	0	0	0	0	0.08 (0.08)	0
*Gempylidae*	*Thyrsites atun*	Barracouta	14	3	20	0.38 (0.37)	0.1 (0.1)	0.11 (0.11)	0	0.75 (0.22)	0
*Gerreidae*	*Parequula melbournensis*	Silverbelly	79	10	31	0	0.5 (0.27)	0	0	5.83 (0.6)	0.67 (0.67)
*Heterodontidae*	*Heterodontus portusjacksoni*	Port Jackson shark	4	1	8	0	0.3 (0.15)	0	0	0.08 (0.08)	0
*Labridae*	*Ophthalmolepis lineolatus*	Southern maori wrasse	1	1	2	0	0	0.11 (0.11)	0	0	0
	*Pseudolabrus rubicundus*	Rosy wrasse	72	13	25	0	1.4 (0.88)	0.67 (0.67)	0	4.33 (1.38)	0
*Lamnidae*	*Isurus oxyrinchus*	Shortfin Mako shark	1	1	2	0.12 (0.12)	0	0	0	0	0
*Latridae*	*Latris lineata*	Striped trumpeter	95	27	22	0	0.2 (0.13)	10.11 (3.47)	0.33 (0.33)	0	0
*Loliginidae*	*Sepioteuthis australis*	Southern calamari	21	3	29	0	0.2 (0.13)	0	0	1.5 (0.23)	0.17 (0.17)
*Monacanthidae*	*Acanthaluteres vittiger*	Toothbrush leatherjacket	15	3	18	0	0	0	0	1.25 (0.28)	0
	*Meuschenia freycineti*	Six-spine leatherjacket	3	1	6	0	0.2 (0.13)	0	0	0.08 (0.08)	0
	*Meuschenia scaber*	Velvet leatherjacket	97	11	53	0	3.3 (0.87)	0.78 (0.43)	0	4.25 (0.97)	1 (0.63)
	*Meuschenia venusta*	Stars and stripes leatherjacket	1	1	2	0	0.1 (0.1)	0	0	0	0
	*Thamnaconus degeni*	Degen's leatherjacket	471	89	41	0	1 (0.42)	0.11 (0.11)	0	36.92 (7.37)	2.83 (1.9)
*Moridae*	*Pseudophycis barbata*	Bearded cod	8	2	12	0	0.1 (0.1)	0.78 (0.28)	0	0	0
*Mullidae*	*Upeneichthys vlamingii*	Southern goatfish	16	8	16	0	0.3 (0.21)	0.11 (0.11)	0	1 (0.65)	0
*Neosebastidae*	*Neosebastes scorpaenoides*	Common gurnard perch	44	4	41	0	1.1 (0.31)	0	0	1.5 (0.42)	2.5 (0.5)
*Ommastrephidae*	*Ommastrephidae sp.*	Squid	9	2	12	0.5 (0.27)	0	0	0.83 (0.4)	0	0
*Ostraciidae*	*Aracana aurita*	Shaw's cowfish	7	2	12	0	0.1 (0.1)	0	0	0.5 (0.19)	0
*Ostraciidae*	*Aracana ornata*	Ornate cowfish	1	1	2	0	0.1 (0.1)	0	0	0	0
*Palinuridae*	*Jasus edwardsii*	Southern rocklobster	2	1	4	0	0	0.22 (0.15)	0	0	0
*Paraulopidae*	*Paraulopus nigripinnis*	Blacktip cucmberfish	47	12	22	2 (0.65)	0.1 (0.1)	0	5 (2.16)	0	0
*Pinguipedidae*	*Parapercis allporti*	Barred Grubfish	36	5	35	2.12 (0.52)	0.8 (0.25)	0.44 (0.44)	0.5 (0.34)	0	0.67 (0.42)
*Platycephalidae*	*Platycephalus bassensis*	Sand flathead	33	6	24	0.12 (0.12)	0	0	0.17 (0.17)	0.83 (0.42)	3.5 (0.96)
	*Platycephalus richardsoni*	Tiger flathead	21	4	25	0	0.2 (0.13)	0	1.67 (0.56)	0.17 (0.17)	1.17 (0.31)
*Pristiophoridae*	*Pristiophorus cirratus*	Longnose sawshark	2	1	4	0	0	0	0	0.08 (0.08)	0.17 (0.17)
*Rajidae*	*Dentiraja lemprieri*	Thornback skate	2	1	4	0	0	0	0	0.17 (0.11)	0
	*Spiniraja whitleyi*	Melbourne skate	9	1	18	0	0.3 (0.15)	0	0.33 (0.21)	0.33 (0.14)	0
*Scyliorhinidae*	*Cephaloscyllium laticeps*	Draughtboard shark	63	4	57	0.12 (0.12)	1.7 (0.5)	0.44 (0.34)	1.33 (0.49)	2.33 (0.31)	0.83 (0.48)
*Sebastidae*	*Helicolenus percoides*	Red gurnard perch	65	12	31	0.25 (0.25)	2.5 (0.81)	4.22 (1.28)	0	0	0
*Serranidae*	*Caesioperca lepidoptera/rasor*	Butterfly and Barber perch	140	60	14	0	6.1 (5.99)	2.11 (1.42)	0	5 (2.79)	0
*Squalidae*	*Squalus acanthias*	Spiny dogfish	1	1	2	0	0	0	0.17 (0.17)	0	0
	*Squalus megalops*	Shortnose spurdog	15	3	14	0.75 (0.49)	0	0	1.5 (0.43)	0	0
*Triakidae*	*Mustelus antarcticus*	Gummy shark	18	2	33	0.25 (0.16)	0	0	1.17 (0.17)	0.25 (0.13)	1
*Triglidae*	*Lepidoperca pulchella*	Tasmanian perch	11	5	10	0	0.1 (0.1)	1.11 (0.56)	0	0	0
	*Lepidotrigla sp*	Gurnards	3	1	6	0	0.1 (0.1)	0.11 (0.11)	0.17 (0.17)	0	0
*Urolophidae*	*Urolophus cruciatus*	Banded stingaree	2	1	4	0	0.1 (0.1)	0	0	0	0.17 (0.17)
	*Urolophus paucimaculatus*	Sparsely spotted stingaree	2	1	4	0	0.2 (0.13)	0	0	0	0
	*Urolophidae sp.*	Stingarees	4	1	8	0.12 (0.12)	0.2 (0.13)	0	0	0	0.17 (0.17)

The Total number of individuals recorded at Max N across all deployments, the greatest MaxN recorded in any single deployment and the prevalence (the percentage of deployments where a species was recorded) are presented as well as the mean (and standard error) MaxN for each assemblage group.

The structure of fish assemblages surveyed was adequately represented by six groups. The overall classification accuracy of the six assemblage groups was high (94%) and a majority of sites (82%) were also assigned to groups with a high degree of confidence (greater than 0.85 probability of membership). Groups four and three were the least certain, but still had a high classification accuracy of 83% and 89%, respectively (Table 2). In addition, these six groups were statistically meaningful with significantly different multivariate community composition (P = 0.001).

**Table 2:** Comparison between the number of sites assigned to each of the six groups using fuzzy clustering and Canonical Analysis of Principle coordinates (CAP).

		CAP Classified Group						Total	Correct
		1	2	3	4	5	6		(%)
Fuzzy Group	1	8	0	0	0	0	0	8	100
	2	0	9	1	0	0	0	10	90
	3	0	1	8	0	0	0	9	89
	4	0	0	0	5	0	1	6	83
	5	0	0	0	0	12	0	12	100
	6	0	0	0	0	0	6	6	100

Jack knife validation in the CAP procedure was used to assess overall and group-wise accuracy of the six groups determined by fuzzy clustering. Classification accuracy of all groups is good, indicating that the groups determined by the fuzzy clustering are robust.

**Figure 2:**
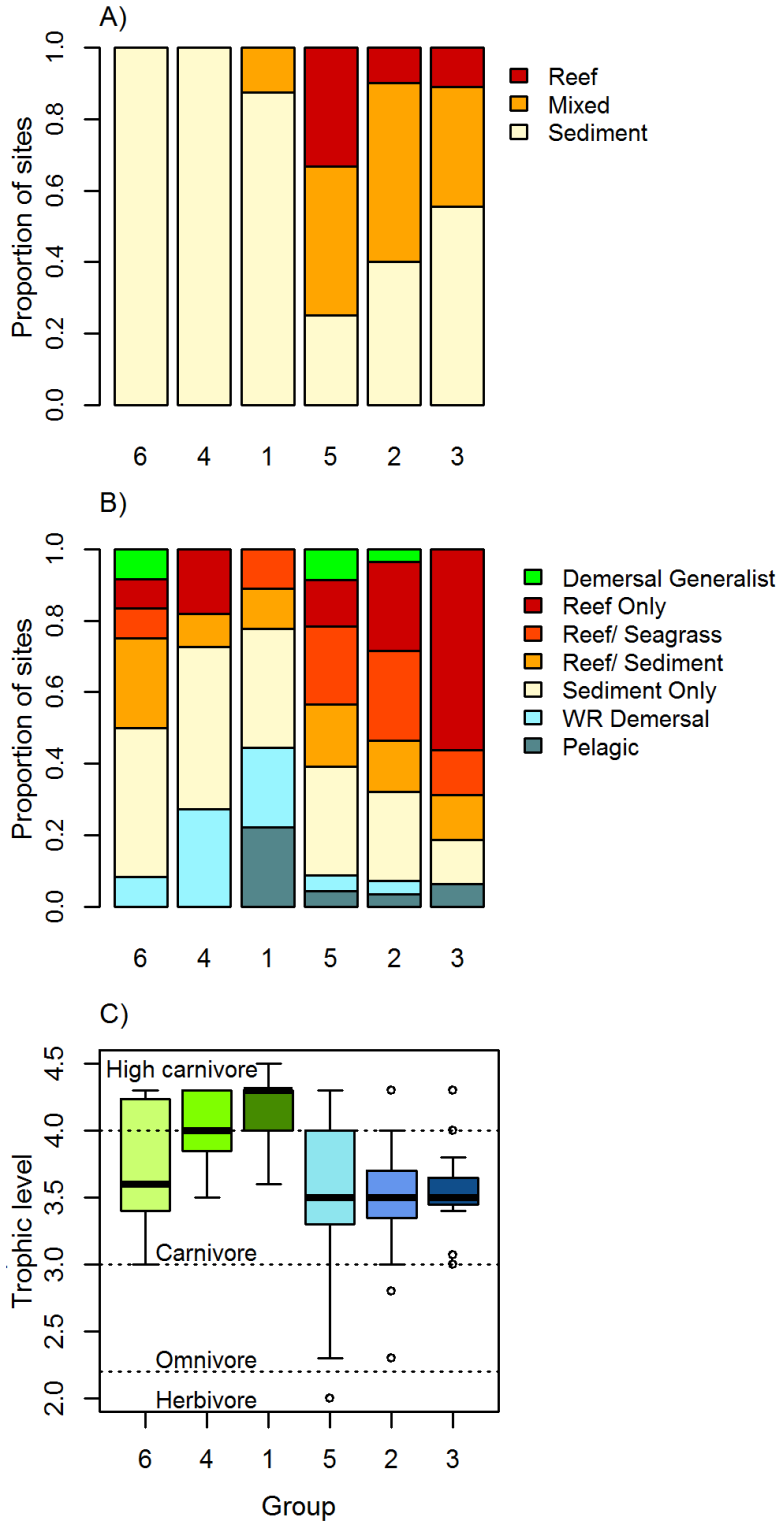
Characteristics of the six assemblage groups identified using fuzzy-clustering. A) Proportion of sites within each group classified as sediment, mixed or reef habitat based on the BRUVs footage; B) the habitat preference of species within each assemblage group; C) boxplot of the trophic level of species within each assemblage group; the box represents the 1^st^ and 3^rd^ quartiles, and circles denote potential outliers. In all panels, groups are ordered based on dominant habitat contained within groups (sediment versus mixed reef/reef) and from shallow to deep within each broad habitat type (based on spatial distribution of groups presented in Fig. 5) In plot C) sediment- associated groups are coloured green and reef-associated groups, blue.

The six assemblage groups were broadly aligned with habitats observed in the BRUV footage. Sites belonging to three groups (groups 1, 4, 6) contained exclusively or predominately sediment habitat (Fig. 2A) and contained greater numbers of species with a preference for sediment habitats. However, species with a strong affinity for reef were also found in the predominantly sediment-dominated habitat group, with groups 4 and 6 containing disproportionately more reef species than suggested by the available habitat (Fig. 2B). Sediment- associated assemblages (particularly groups 1 and 4) also contained a relatively high proportion of wide-ranging demersal species. Groups 2, 3, and 5 contained moderate to high proportions of mixed reef and reef habitat with the highest proportion of reef-associated species observed in group 3 (Fig. 2A, B).

Species representing a range of trophic levels were recorded in BRUV footage. Carnivores were the most numerous species, as expected given the use of baits. Even so, the average trophic level of species differed between the six assemblage groups (df = 5, F = 5.46, P<0.001; Fig. 2C). Groups 1 and 4 (sediment groups) contained species with significantly higher trophic level (higher carnivores) than groups 2 and 3 (mixed-reef) which contained primarily carnivores. The remaining two groups (5 and 6) contained species from a relatively wider range of trophic levels (Fig. 2C) with group 5 represented by the largest range in values and the only herbivore observed.

**Figure 3:**
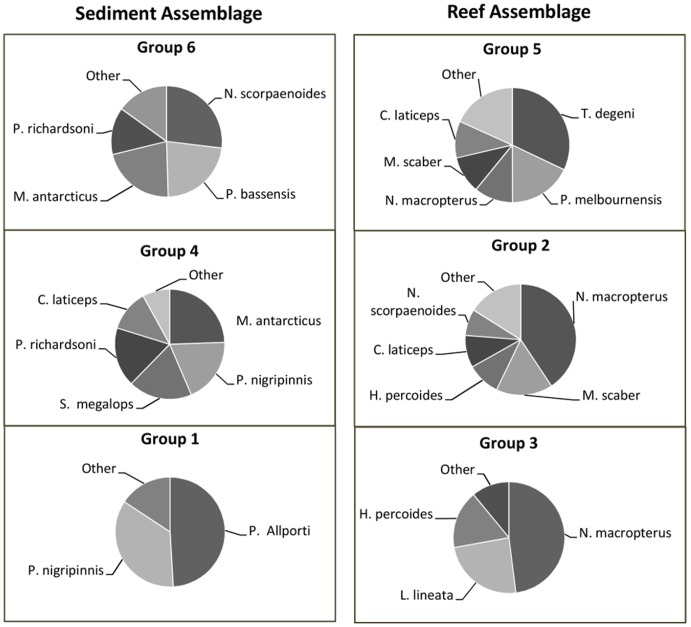
Species contributing up to 80% towards the similarity of the predominantly sediment- and predominantly reef- associated groups identified using fuzzy clustering.

The characteristic species of each assemblage group (making the highest contributions to within-group similarity) re-enforced the broad habitat-related patterns described above (Fig. 3). Sediment-associated species such as flathead (*Platycephalus bassensis*, *Neoplatycephalus richardsoni*) and sharks (*Mustelus antarcticus, Squalus megalops*) as well as the common gurnard perch (*Neosebastes scorpaenoides*) and cucumber fish (*Paraulopus nigripinnis*) were characteristic of groups 4 and 6. Group 1 also consisted of sediment-associated fish (eastern- barred grubfish - *Parapercis allporti-* as well as *P. nigripinnis*), but was differentiated from groups 4 and 6 by relatively low abundances and very few species. Of the predominantly mixed-reef associated groups, groups 2 and 3 were both dominated by jackass morwong (*Nemadactylus macropterus*). Group 2 was also composed of a variety of others species including the velvet leatherjacket (*Meuschenia scaber*), reef ocean perch (*Helicolenus percoides*) and draughtboard sharks (*Cephaloscyllium laticeps*). Group 3 was further characterised by relatively high abundances of striped trumpeter (*Latris lineata*) as well as reef ocean perch. Group 5 was differentiated from other groups by large numbers of leatherjackets (primarily *Thamnaconus degeni*), but was also characterised by silverbelly (*Parequula melbournensis)* and jackass morwong.

### Relationships with environmental variables

**Figure 4:**
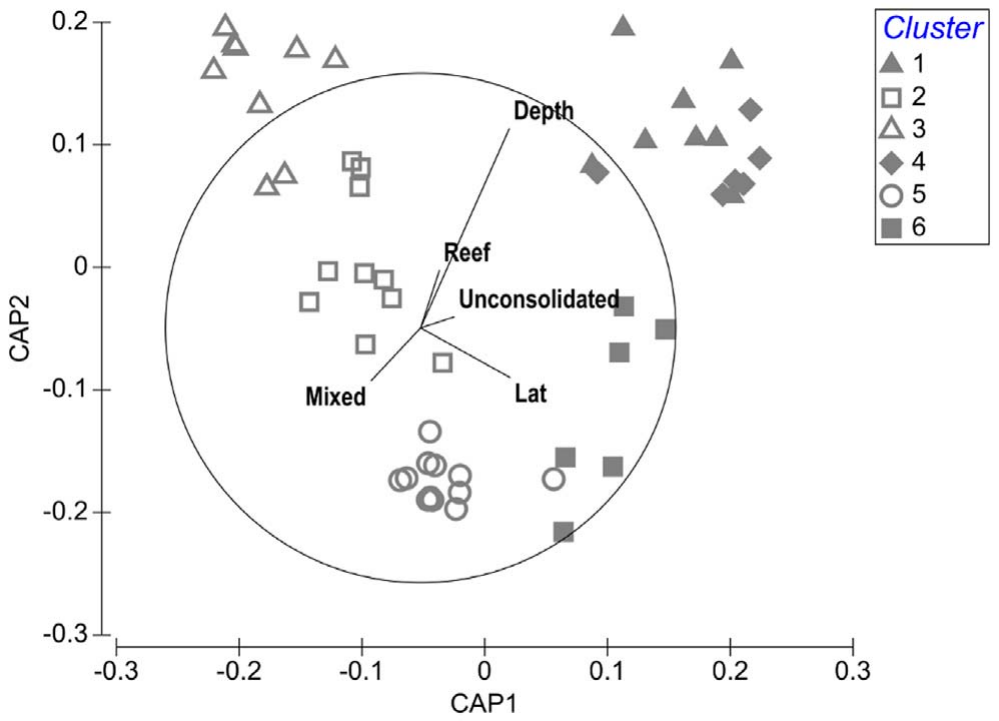
Canonical Analysis of Principle coordinates (CAP) plot discriminating sites based on fuzzy clustering. Each site was assigned to the cluster for which it had the highest probability of membership. Sites are shown as symbols: filled grey symbols represent sand-associated assemblages and open symbols represent reef-associated assemblages. Vectors of all environmental variables are overlaid and are proportional to their correlation with either CAP axis one or two.

Environmental factors correlated well with the fish assemblage groupings (Fig. 4). Depth delineated groups most strongly (a correlation with CAP axis 2 of 0.78) and groups 1, 4 and 3 were generally found at greater depth. Substratum type also influenced assemblages, with sediment-associated groups (1, 4 and 6) falling on the right hand-side of the CAP plot, confirming the patterns seen in the composition of species above. Latitude was also moderately correlated with axis one (0.35) and appears to separate the mixed/reef associated groups 2, 3 and 5.

**Figure 5:**
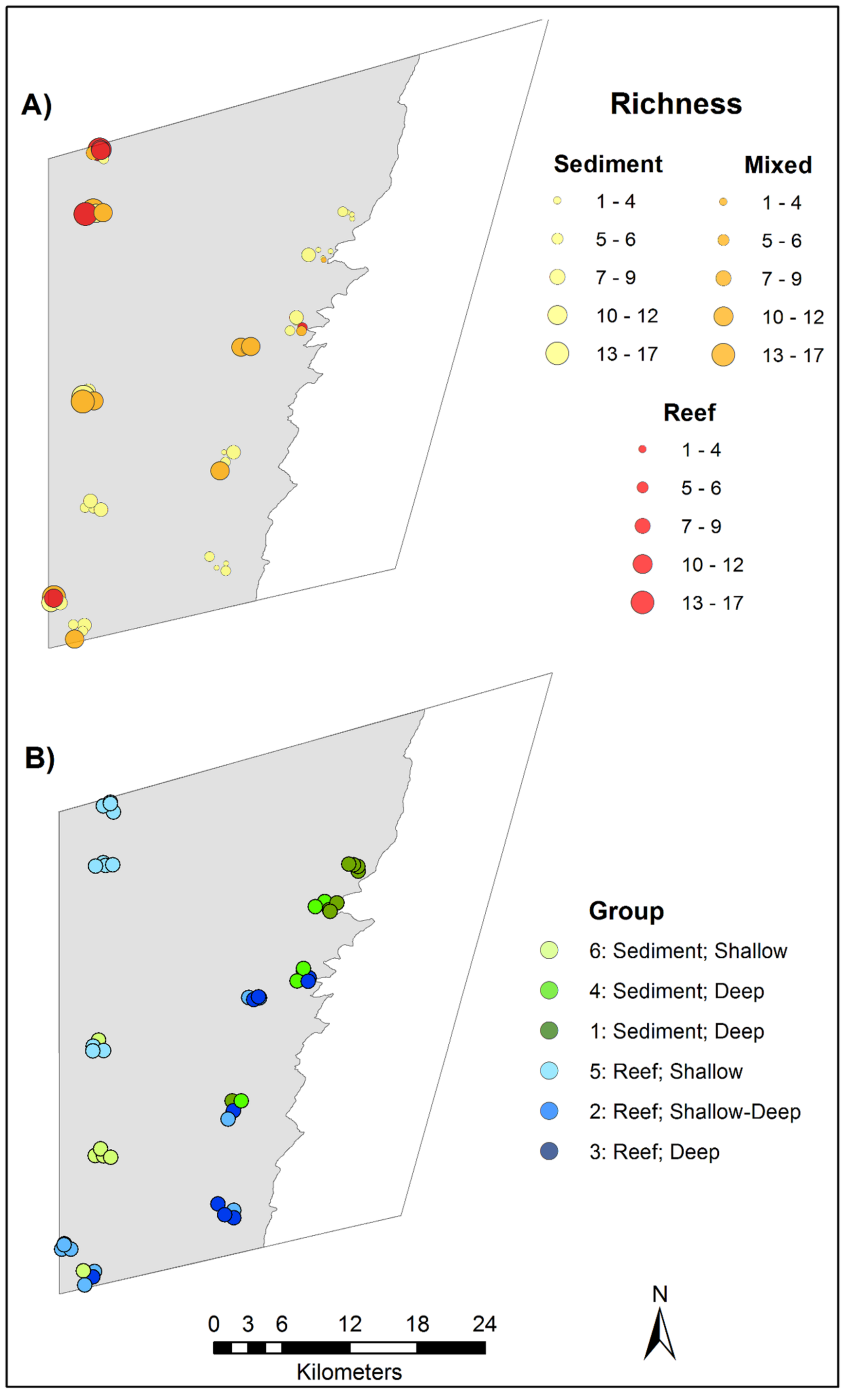
Spatial distribution of A) species richness B) fish assemblage groups on the Flinders CMR shelf (shaded grey). Increasing size of symbols in A) indicate increasing species richness. Symbols are colour coded according to the observed substratum type in BRUV footage: yellow =  sediment; orange =  mixed; red =  reef. Assemblages in B) are coded by colour, with predominantly sediment-associated assemblages coloured green and predominantly reef-associated assemblages coloured blue.

Species richness was also correlated with environmental factors. Richness decreased with depth and mixed and reef substratum also supported more species than sediments (Table 3). Latitude however, was not correlated with species richness (Table 3). This resulted in spatial patterns as depicted in Fig. 5, where assemblages are generally richer towards the shallow, western side of the reserve.

**Table 3:** Results of Poisson GLMM for species richness recorded in BRUV deployments.

Factor	Co-efficient	Std Error	df	t-value	p-value
Depth	−0.277	0.067	36	−4.1489	0.0002
Mixed Habitat	0.330	0.092	36	3.575	0.0010
Reef Habitat	0.382	0.121	36	3.169	0.0031

Predictor variables have been scaled and centered and co-efficients are on the scale of the link function (log). Spatial clusters introduced as part of the sampling design were included as a random effect and the effects of habitat type (sediment, mixed or reef) are presented relative to sediment habitat.

### Spatial distribution of fish assemblages across CMR

Fish assemblages showed distinct spatial patterns across the Flinders CMR (Fig. 5). When combined with depth, the assemblages can be broadly categorised as: shallow, reef-associated (group 5); intermediate reef-associated (group 2); deeper, reef-associated (group 3); shallow sediment-associated (group 6); and deeper, reef-associated (groups 1 and 4). While sites within the same spatial cluster often belonged to the same fish assemblage, this was not always the case. Clusters of sites close to the shelf edge and in the south of the reserve were often more heterogeneous, composed of two to three different assemblages within a 1 km radius.

## Discussion

This study trialled the use of BRUVs and a spatially- balanced survey design to provide the first quantitative description of demersal fish species and assemblages occurring the multiple use zone of the Flinders CMR shelf. Non-extractive sampling with BRUVs identified characteristic species (including jackass morwong, leatherjackets, draughtboard sharks, reef ocean perch and common gurnard perch), several species of commercial interest (including gummy sharks, reef ocean perch, striped trumpeter and jackass morwong) and species from trophic groups ranging from omnivores through to higher predators. While many of the species present in the study area are widely distributed across southern and/or south-eastern Australia, the majority are endemic to Australian waters. This highlights the unique biodiversity represented in temperate Australia, primarily as a result of its relatively isolated evolutionary history [39], [40], and reinforces the global importance of conserving representative fauna.

### Spatial and environmental patterns in fish assemblages

Six assemblages with a distinct spatial pattern were observed at sites across the reserve. Many species contributed to more than one assemblage and differences in the relative abundance of species defined the differences between several assemblages. Assemblages were structured by depth and habitat type, and could be heterogeneous at relatively small scales (i.e. within a sampling cluster).

Depth-related assemblage transitions were most distinct and occurred in waters greater than approximately 80 m for sediment-associated assemblages and approximately 60 m for reef-associated assemblages, which is largely consistent with other studies on the southeast Australian shelf [41]. While some species were found across almost the full depth range of the study area (e.g. jackass morwong and the flathead, *Platycephalus richardsoni*) several species characterised particular substratum and depth zones. Leatherjackets *(T. degeni* in particular) for example, were characteristic of shallow reef sites; striped trumpeter were indicative of deeper, reef-associated assemblages; and cucumber fish were indicative of deeper, sediment-associated assemblages. These results accord with other studies where depth has a major influence on the distribution of fish assemblages [17], [42].

As well as influencing assemblage composition, depth also influenced the diversity and ecology of assemblages. Species richness generally declined with depth consistent with observations elsewhere [39], [43], [44] and the trophic range of species occurring in shallow assemblages was generally greater than deeper assemblages. In reef-associated assemblages for example, larger trophic ranges of the shallow water assemblage were due to the co-occurrence of species such as leatherjackets (many of which are omnivorous) and draughtboard sharks (which are carnivores). Similarly, other studies have noted trophic-related changes with depth, such as increases in body size [42] and a transition towards higher –order predators [34].The combination of assemblage level patterns and changes in ecology and diversity with depth as seen here, have been used to suggest that the edge of the continental shelf forms an important faunal break. Consequently the shelf and slope environments should be considered separate management units [8], [31] and not subsumed within the same management zone as currently occurs within the Flinders CMR.

Although a ubiquitous pattern, the processes involved in the depth structuring of benthic fish assemblages can be varied. In the shelf environments of the Flinders CMR, we suggest these are partly attributable to the strong tidal currents which affect the shallow, western section on the reserve [22]. In this region, low-profile reefs are dominated by tall biogenic structures with flexible forms such as sea whips and erect branching sponges (personal observation) that are indicative of high energy environments. This 3D biogenic structure is much reduced towards the deeper, eastern extent of the study area. These differences in reef structure [45], as well as physical limitations posed by increased current velocities [46], may have a large influence on the assemblages found in shallow regions of the reserve. Other physical and oceanographic conditions also co-vary with depth. For example, light availability decreases with depth, which affects primary productivity and therefore the range of food sources available to fish and appears to correspond with the observed changes in the trophic composition of assemblages. In addition, the oceanography in this region is complex, driven largely by inter-annual and inter-seasonal variability in the southward penetration of the warm-water, East Australian Current (EAC). The EAC can incur on the shelf or form fronts at the shelf break [24] and affects processes such as recruitment [47] and primary productivity [48] that are in turn likely influence the composition of fish assemblages, particularly on the outer shelf. Regardless of the proximal processes involved, the depth structuring of assemblages observed in the study area suggests that future sampling would benefit by breaking the region into shallow and deep strata around the 70 m depth mark.

Habitat or substratum type was also influential in discriminating between the assemblages observed in the Flinders CMR shelf. Despite preferentially sampling clusters of sites surrounding our initial mixed/reef sites, a large number of all sites surveyed contained only sediments, at least within the field of view of the BRUVs. This occurred because low-profile shelf reefs in the CMR are small scale features (tens of meters) and their distribution is highly patchy. Sediment sites were generally less species rich than those found on mixed or reef substrata. However, the composition of these sites varied enough to form three distinct assemblage groups, primarily on the basis of their depth distribution. The lower diversity of sediment assemblages has been attributed to lower habitat complexity with fewer niches to support co-existing species [49]. Still, sediment-associated assemblages contained some species with affinity for reef habitats. This may be because reef habitat was present outside the BRUVs field of view but within the swimming distance of fishes resulting in a ‘halo’ effect where species with an affinity for reef and sediments co-occur [49]. In subtropical shallow reefs this halo can extend 200 m from the reef edge [49]. The importance of local substratum in structuring fish assemblages highlights the need to develop comprehensive, high resolution habitat maps for the reserve. Assembling these habitat maps for marine reserves will require a commitment to strategic mapping of the seabed using multibeam sonar, so that over time complete coverage is achieved. For the continental shelf, this mapping could take years to decades [50]. In the meantime, management will need to adopt spatially representative sampling such as presented in this study to provide habitat and biodiversity inventories and to underpin monitoring programs.

Geomorphic features of the seafloor, such as submarine canyons, can substantially alter water flows, enhance local productivity [51] [52] and influence fish assemblage patterns [53]. Canyons are a conservation feature of the Flinders CMR and several canyons intersect the shelf edge. Canyons were represented in our sampling by a cluster of sites near a canyon head in the north of the survey area. Assemblages adjacent to canyon heads were not however, unique to canyon heads and were also found in other deeper sections of the shelf. Neither were these assemblages particularly species rich. Despite our preliminary findings, a better understanding of the ecological significance of canyon heads in this system will require a more targeted and intensive sampling strategy within and around the canyon head systems, e.g. [51].

### Implications for Monitoring and Management

An objective of the Flinders CMR – and the larger Southeast Marine Reserve Network of which it is part - is to protect the biodiversity, natural and cultural values of the region. These include “representative examples of the ecosystems, communities and habitats” associated with the Tasmanian Shelf and Southeast Shelf Transition Provinces [21]. While these values are defined in broad terms from the national bioregionalisation, these have yet to be translated into inventories of conservation values, with specific metrics for monitoring their status. This study is a first step towards developing both an inventory of biological values, and elements of an effective biological monitoring program, including relevant environmental influences, for fish communities represented in the shelf component of the Flinders CMR. We suggest that representative demersal fish communities include those found on dynamic, low- profile reefs in both shallow (<70 m) and deep (70–160 m) areas of the shelf as well as those found on sediments in the same depth strata, and here we have characterised each of these assemblages. Several prevalent commercial and recreational species, including striped trumpeter and jackass morwong, which are likely to be responsive to management actions may be good candidates as indicator species and this work will provide the basis for further research into deriving specific monitoring targets.

We used a non-standard sampling design, GRTS, to survey fish assemblages and to satisfy the objectives of the broader survey of the Flinders CMR. Due to the cost and logistics associated with sampling in offshore and/or remote environments, the use of survey designs that are efficient, representative and flexible enough to accommodate multiple objectives is likely to become increasingly important. This is particularly true when simultaneously considering the inventory and monitoring needs of multiple reserves within networks. In the present study, the GRTS sampling ensured that clusters of reef and sediment sites sampled with BRUVs are well spread out across the reserve (within the distribution of each habitat type) and consequently encompass broad spatial and environmental gradients. This in turn provides confidence in the patterns observed and provides a better basis for additional or repeated sampling in a monitoring context. The site-ordered sampling of GRTS has the potential disadvantage of using ship time inefficiently if there is a large cumulative distance between deployments. This would have been the case in our study, but was circumvented by clustering samples around a subset of original sites. Conversely, the site ordering of GRTS allows dynamic altering of samples according to situations in the field, while maintaining spatial balance within strata.

The GRTS methodology will also be useful when choosing samples for future sampling, typically as part of an adaptive monitoring program. The master list of samples, that encompasses all possible sampling locations [28], can be subset in different ways by varying the inclusion probabilities for factors such as habitat types or depth, to accommodate new research questions or changing management objectives. While such changes lead to higher or lower sampling rates for different habitats or strata, the set of sites selected always maintains spatial balance within the strata. Inclusion probabilities are taken into account when deriving representative estimates of the occurrence, proportions or abundance of metrics or indicators across the entire sampling area [18]. Once suitable indicator species and metrics have been selected, the GRTS master sample can provide the basis for rotating panel designs [28] for repeat sampling that aims to detect changes in the abundance of these species, and possibly also major shifts in the distribution of assemblage types. Another advantage of GRTS in this respect, is its use of a local neighbourhood variance estimator, which is generally unbiased and more precise than variance estimators from other types of sampling designs [26]. Future sampling within the context of monitoring however, will need to incorporate comparable areas outside of the multiple use management zone of the Flinders CMR. In addition sufficient sample sizes will be needed to provide enough power to distinguish between natural variability and biological trends in order to be able to assess whether current management controls are effective in achieving their stated objectives.

The use of baited systems to survey fish communities has sometimes been criticised because of the bias towards sampling scavenging and predatory species [54]. However, all fish sampling methods have selectivity biases, and alternatives for sampling deep continental shelf fishes (e.g. trawls, meshnets and traps [41]) are extractive (and some destructive to the habitats the reserve is intended to protect). We recognise that by using BRUVs we have preferentially observed a subset of species that is attracted to, or undeterred by, baits. However, BRUVs data have been shown to clearly discriminate between fish assemblages in a variety of environmental settings [43] and have better statistical power to detect spatial and temporal changes in assemblage structure and abundances of individuals than unbaited methods [54]. In the future, we may complement BRUVs with other non-extractive video techniques (e.g. forward looking cameras on remotely or autonomously operated vehicles) to capture other components of the community. Never-the-less, in our present study BRUVs successfully observed a broad range of species, including key ecological species and potential indicator species. Standardised methodologies, in combination with robust sampling designs, will be essential for ongoing monitoring and assessment across multiple reserves in Australia's reserve network. Finally, the challenges we outline for the inventory and long-term monitoring of large and remote marine reserves are not unique to the Australian reserve network, nor exclusively to marine reserves. Therefore the approach that we have demonstrated may be usefully applied in other systems and under other management scenarios.

## Supporting Information

Figure S1 **The location of the forty GRTS sites sampled across the Flinders CMR shelf in phase one.** Sites are coloured according to the broad habitat type: sediment (yellow); mixed, low-profile reef and sediments (red); canyon head (blue) recorded during phase one of sampling. Sites are labelled with their GRTS site number which, when sampled in order, represents a spatially balanced sample. The table shows the subset of sites sampled as the basis of clusters in the phase two sampling with BRUVs. These sites are the first three GRTS sites classified as sediment and the first eight classified as mixed reef.
(PDF)

Data S1 **Matrix of the species observed at each BRUV deployment.** Environmental variables used in analyses are also included.
(XLSX)

Data S2 **Attributes of species used in analysis, including trophic level, habitat preference and endemicity.** Summary statistics on the prevalence and abundance of species across the survey area are also given.
(XLSX)
