# Supplementary material for: Quantifying Fish Assemblages in Large, Offshore Marine Protected Areas: An Australian Case Study
Source: PLoS One. 2014 Oct 31;9(10):e110831. doi: 10.1371/journal.pone.0110831 (PMC4215995; doi:10.1371/journal.pone.0110831)
Supplement: Figure S1 — The location of the forty GRTS sites sampled across the Flinders CMR shelf in phase one. Sites are coloured according to the broad habitat type: sediment (yellow); mixed, low-profile reef and sediments (red); canyon head (blue) recorded during phase one of sampling. Sites are labelled with their GRTS site number which, when sampled in order, represents a spatially balanced sample. The table shows the subset of sites sampled as the basis of clusters in the phase two sampling with BRUVs. These sites are the first three GRTS sites classified as sediment and the first eight classified as mixed reef. (PDF) [file pone.0110831.s001.pdf]

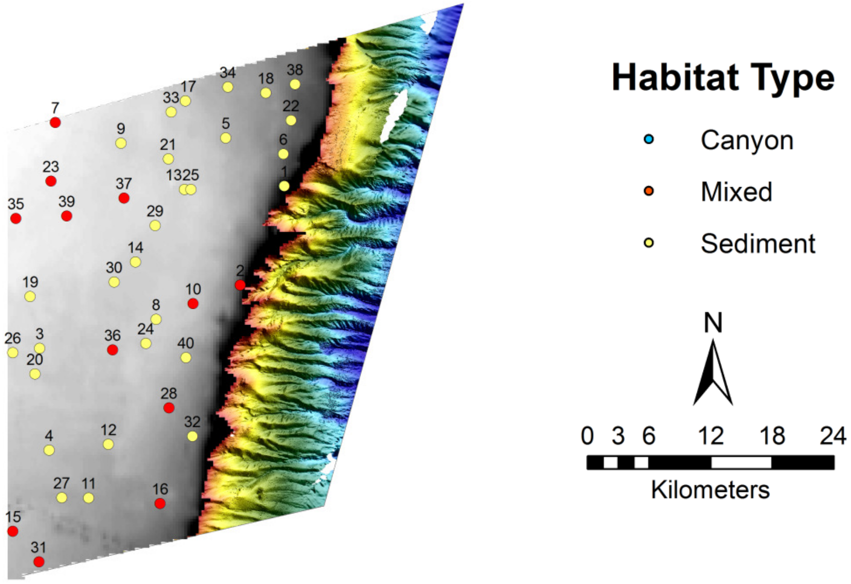


| Phase 1 GRTS Sample Site | Seabed Habitat Class | Depth (m) |
|---|---|---|
| 1 | Sediment | 140 |
| 3 | Sediment | 59 |
| 4 | Sediment | 70 |
| 2 | Mixed | 160 |
| 7 | Mixed | 40 |
| 10 | Mixed | 65 |
| 15 | Mixed | 68 |
| 16 | Mixed | 98 |
| 23 | Mixed | 45 |
| 28 | Mixed | 83 |
| 31 | Mixed | 79 |
